# Angle–torque relationship and muscular activity patterns during concentric contractions with a novel variable cam system

**DOI:** 10.3389/fspor.2026.1913115

**Published:** 2026-09-29

**Authors:** Renato da Costa-Machado, Diogo L. Marques, Runer A. Marson, Letícia Costa de Sousa, Hugo Plácido da Silva, Hugo Louro, Daniel A. Marinho, Ana Conceição

**Affiliations:** 1 Sport Sciences School of Rio Maior, Polytechnic Institute of Santarém, Rio Maior, Portugal; 2 Department of Sport Sciences, University of Beira Interior, Covilhã, Portugal; 3 Research Centre in Sports, Health and Human Development (CIDESD), Covilhã, Portugal; 4 Brazilian Army, Preparatory School for Army Cadets, Campinas, SP, Brazil; 5 Department of Bioengineering, Instituto Superior Técnico, Lisboa, Portugal; 6 Instituto de Telecomunicações, Lisboa, Portugal

**Keywords:** moment arm, muscle activity, resistance training, strength curves, torque, variable-cam

## Abstract

**Introduction:**

Understanding the angle -torque relationship requires recognizing that torque can differ across contraction modes. This study aimed to analyze the performance of the Variable Moment Arm Cam® (VMAC®) by examining torque production and muscle activation during concentric leg extension contractions across the full range of motion (ROM) (100°–0°) and comparing them with those obtained using an isokinetic dynamometer.

**Methods:**

In total, 22 young men (20.55 ± 2.19 years; 175.41 ± 7.21 cm; 72.80 ± 9.90 kg; 18.00 ± 5.83% body fat; 23.63 ± 2.82 kg·m⁻^2^) participated in four randomized moments of torque and muscle activity assessments, two using the VMAC® and two using an isokinetic dynamometer. Torque and muscle activity data were synchronized and recorded across the entire ROM (100°–0°) and evaluated at 5° intervals.

**Results:**

The VMAC® system produced strength curves that closely matched those observed when using the isokinetic dynamometer across the initial and mid-ROM joint angles (100°–35°), peaking around mid-ROM (70°–65°). Muscle activity differed across conditions while the torque production remained similar.

**Conclusion:**

The VMAC® generated torque patterns comparable to isokinetic dynamometry, showing the strongest alignment at mid-ROM, supporting the use of this system to replicate natural human strength curves while highlighting the opportunity to refine cam geometry.

## 1 Introduction

Understanding how torque is produced throughout the range of motion (ROM) is fundamental for interpreting human strength capabilities and optimizing resistance training prescription (1–4). Joint angle-specific torque profiles influence the mechanical stimuli and neuromuscular activation that underpin strength and hypertrophy adaptations (3). Despite this importance, the majority of traditional resistance machines apply a constant external load, which does not reflect the non-linear nature of human strength curves and may limit the ability to train muscles effectively across the full ROM (5, 6).

To address this mismatch, variable resistance systems, such as variable cam systems (7, 8), have been developed to better align external resistance with the user's physiological angle–torque behavior (9–12). Although these systems are conceptually designed to match human torque capability (HTC), experimental findings consistently show that machine resistive torque (MRT) profiles still diverge from the actual shape of human strength curves. Variable cam systems are often presented as a way to modulate resistance in line with theoretical force-production models, yet evidence from several studies (7, 8, 12, 13) demonstrates that their real-world behavior rarely mirrors physiological demands.

Isokinetic dynamometry is widely regarded as the gold standard for assessing torque production across the ROM (14, 15). Its controlled angular velocity, high measurement precision, and strong repeatability make it an ideal reference for evaluating new resistance systems. However, isokinetic devices are expensive, require specialized expertise, and lack ecological validity for typical training environments, which emphasizes the interest in alternative systems capable of providing accurate, angle-specific resistance in more accessible and training-relevant contexts.

Recent advances in sports technology have led to the development of new resistance machines that integrate mechanical cams with digitally controlled resistance profiles (16, 17). Among these innovations is the Variable Moment Arm Cam® (VMAC®), which modulates torque through a system of 18 evenly spaced radial pins, positioned at 20° intervals around the rotational axis, thereby configuring several moment arm length adjustments (18).

To characterize the angle–torque relationship in the leg extension exercise, Folland et al. (11) assessed the isometric peak torque (PT) at four joint angles between 110° and 50° of knee flexion, reporting an inverted U-shaped curve peaking near 70° with the lowest value at 110° (71% PT, approximately). In another study assessing angle–torque relationship during concentric contractions, Folland and Morris (12) identified the PT at 80°–60°. However, comparisons with other equipment revealed consistent mismatches, differing significantly from the isokinetic dynamometer torque assessments at 180°/s and 90°/s. This is further illustrated by the muscle activation discrepancies reported by Häkkinen et al. (1), who demonstrated load-dependent muscle activity patterns during concentric actions, with stable or slightly rising activation at 100% repetition maximum (RM) load and decreasing profiles at 80% RM and 60% RM. Aboodarda et al. (19), examining concentric–eccentric actions from 100° to 0°, reported progressively increasing activation across the ROM, from 39.88 to 114.37 mV·s. This was similar to the results later obtained by Aboodarda et al. (20), who described a progressive increase from approximately 41% to 91% maximum voluntary contraction (MVC). Both studies used a Nautilus leg extension with added elastic bands.

In a previous study analyzing the use of the VMAC® system for maximum isometric contractions, the equipment demonstrated torque and muscle activity profiles that closely resembled isokinetic dynamometer patterns, with strongest validity at 60° (standard error of estimate: 0.68%), where the torque peaked for both pieces of equipment (99.82 ± 0.45% PT for the VMAC® and 99.80 ± 0.60% PT for the isokinetic dynamometer), while variability increased at ROM extremes [coefficient of variation (CV): 17.17%–33.44%], indicating good HTC replication but highlighting the need for continued cam design refinement (18).

Understanding the angle–torque relationship requires acknowledging that torque measured under isometric conditions does not always mirror the patterns observed during dynamic contractions and may vary. Differences in contraction mode, velocity, and neuromuscular strategy can lead to distinct torque–angle profiles, making it essential to interpret isometric and dynamic assessments with caution. This highlights the need to further evaluate the VMAC® under different conditions, namely concentric contractions. Considering this, this study aimed to analyze the performance of the VMAC® by examining torque production and muscle activation during concentric leg extension contractions across the full ROM (100°–0°) and comparing them with those obtained using an isokinetic dynamometer. The following four hypotheses were formulated: (i) the torque values produced by the VMAC® would closely align with those obtained from the isokinetic dynamometer across the ROM, reflecting similar torque–angle profiles; (ii) the torque values obtained with the VMAC® would replicate the inverted U-shape strength curves observed in the isokinetic dynamometer across the ROM, indicating comparable torque–angle behavior between systems; (iii) the torque measurements collected with the VMAC® would show low between-session variability, supporting its within-device reliability; and (iv) despite similarities in torque output, the muscle activation patterns recorded with the VMAC® would differ from the muscle activity patterns observed when using the isokinetic dynamometer due to differences in loading intensity.

## 2 Materials and methods

### 2.1 Experimental approach to the problem

This study used a repeated-measures, within-subject laboratory design to evaluate whether the VMAC® system could reproduce torque and load-dependent muscle activation patterns comparable to those obtained with an isokinetic dynamometer. The participants completed four testing sessions that were separated by 72–96 h to minimize fatigue and ensure consistent recovery. Each participant performed two sessions on the VMAC® and two on the isokinetic dynamometer, enabling direct comparison between devices while controlling for individual variability. To test the hypothesis that the VMAC® can modulate resistance throughout the ROM, torque and muscle activity were recorded continuously from 100° to 0° of knee flexion. Measurements were taken at 5° intervals, providing high-resolution angle-specific profiles for both dependent variables. Torque was the primary mechanical outcome because it directly reflects force production across joint angles. Muscle activity was included as a complementary outcome to determine whether neuromuscular demands changed across conditions, thereby assessing whether resistance profiles were aligned with physiological capacity. This design allowed for a rigorous evaluation of the VMAC®'s ability to approximate dynamometer-derived torque patterns.

### 2.2 Subjects

In total, 22 healthy young men participated in the study (20.55 ± 2.19 years; 175.41 ± 7.21 cm; 72.80 ± 9.90 kg; 18.00 ± 5.83% body fat; 23.63 ± 2.82 kg·m⁻^2^). All the volunteers provided written informed consent prior to involvement. No specific physical activity level was required for inclusion. Participants were excluded if they (i) had any lower- or upper-limb injury limiting physical activity within the previous 6 months, (ii) had undergone surgery on the lower or upper limbs in the past 12 months, (iii) had a medical condition that could impair proper test performance, or (iv) used medication known to influence strength (15). The study protocol received approval from the University of Beira Interior Ethics Committee and complied with the principles of the Declaration of Helsinki for research involving human participants (CE-UBI-Pj-2025-008).

### 2.3 Procedures

#### 2.3.1 Equipment

Trials were performed on an isokinetic dynamometer (Humac Norm, CSMi, USA) to calculate the participants’ HTC and on the novel variable cam system to calculate its MRT.

The VMAC® is a structurally robust and modular system capable of precise torque modulation for biomechanical applications with a framework consisting of two steel plates with steel pins that act as adjustable cable contact points, enabling fine control of the effective moment arm. This system allows 10 discrete pin positions, producing a moment arm adjustment range of 30–300 mm relative to the axis of rotation. The VMAC® system was integrated into a leg-extension machine using a chamfered circular shaft aligned with the pin placement row, where a cable connected the VMAC® to a load cell. An adjustable seat with an 85° backrest ensured alignment between the knee and cam axes. The complete system measured approximately 1,490 mm × 1,200 mm × 1,370 mm (Figure 1).

**Figure 1:**
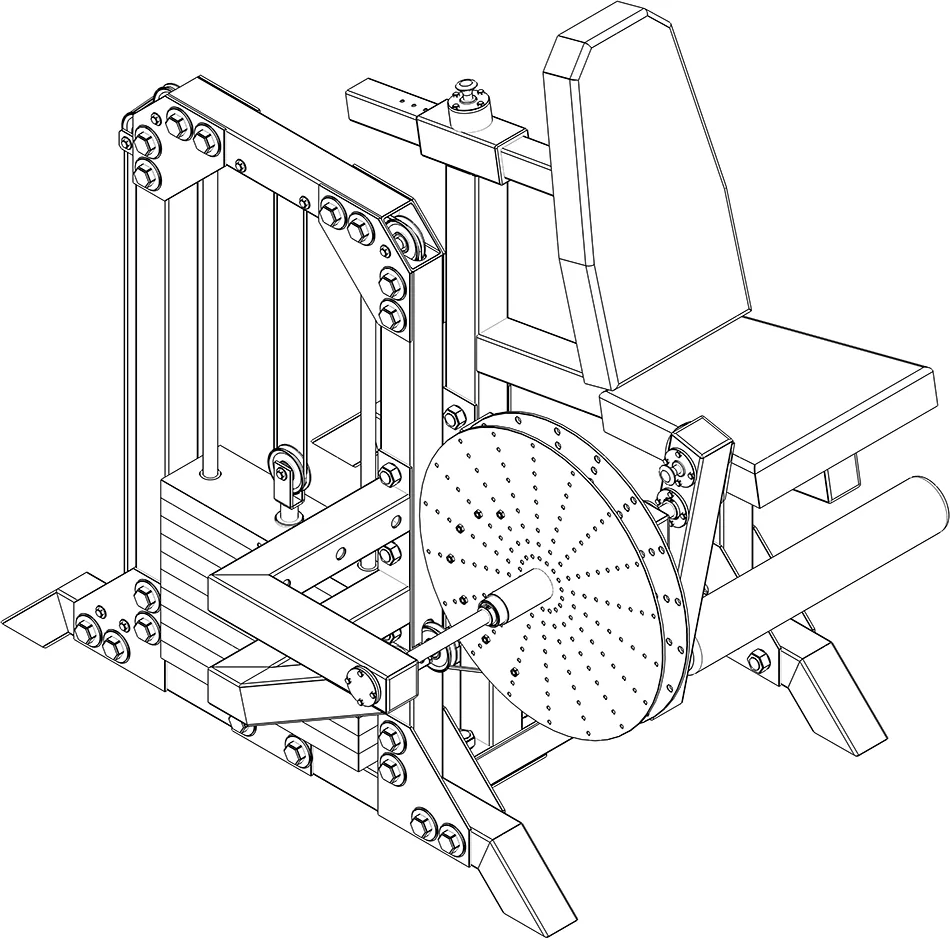
Integration of the VMAC® into the leg extension machine. Adapted from da Costa-Machado et al. (18).

Surface electromyography (sEMG) was conducted using a Biosignals Plux system (Plux, Lisbon, Portugal) equipped with sensors featuring a 10-mm inter-electrode distance, and acquired using Monitor Plux (Plux, Lisbon, Portugal). Torque and sEMG data were obtained from the isokinetic dynamometer for HTC and the VMAC® system for MRT. The torque on the dynamometer was captured using HUMAC2015 software (Humac Norm, USA), whereas torque data from the VMAC® were collected with Chronojump version 2.3 (Boscosystem, Barcelona, Spain).

#### 2.3.2 Testing

The participants visited the laboratory 2 weeks before the first testing session to familiarize themselves with the equipment and to determine electrode placement sites. Before data collection, the participants completed an anamnesis questionnaire covering date of birth, sex, preferred/dominant lower limb for force production, musculoskeletal injury and medical history, medication use, and device-specific considerations relevant to bioelectrical impedance analysis. Subsequently, they were introduced to the testing procedures and underwent anthropometric assessment, including height measurement and weight and fat mass evaluations via bioelectrical impedance analysis using an InBody 720 (Biospace Co., Ltd., Seoul, Republic of Korea). The participants then performed three submaximal and two maximal familiarization repetitions (21) of the leg extension movement on a Humac NORM isokinetic dynamometer (Computer Sports Medicine, Inc., Stoughton, MA, USA) and the VMAC® system.

Following familiarization, the participants underwent four evaluation sessions spaced 72–96 h apart, consisting of two HTC and two MRT assessments, counterbalanced through randomized ordering. This recovery window was selected to minimize residual fatigue while avoiding meaningful fluctuations in strength capacity (22). On each testing day, the participants began with a standardized warm-up consisting of 5 min of cycling at 100 W and 60 rpm on a Monark Ergomedic 828E ergometer (Monark Exercise AB, Vansbro, Sweden) (23). Each participant performed two sessions under the HTC condition and two under the MRT condition (Figure 2). The HTC assessment consisted of three randomized maximum concentric contraction sets at different angular velocities (60, 90, and 180°/s) and the MRT assessment consisted of three randomized concentric contraction sets with different loads (40, 30, and 20 kg). All sets consisted of five repetitions, and both the non-dominant side (NDS) and dominant side (DS) were assessed randomly. To ensure adequate neuromuscular recovery and prevent fatigue accumulation, a 3-min rest interval was provided between all trials to ensure adequate neuromuscular recovery and avoid the appearance of fatigue. During all sessions, torque and muscle activity were assessed at 5° intervals across the ROM.

**Figure 2:**
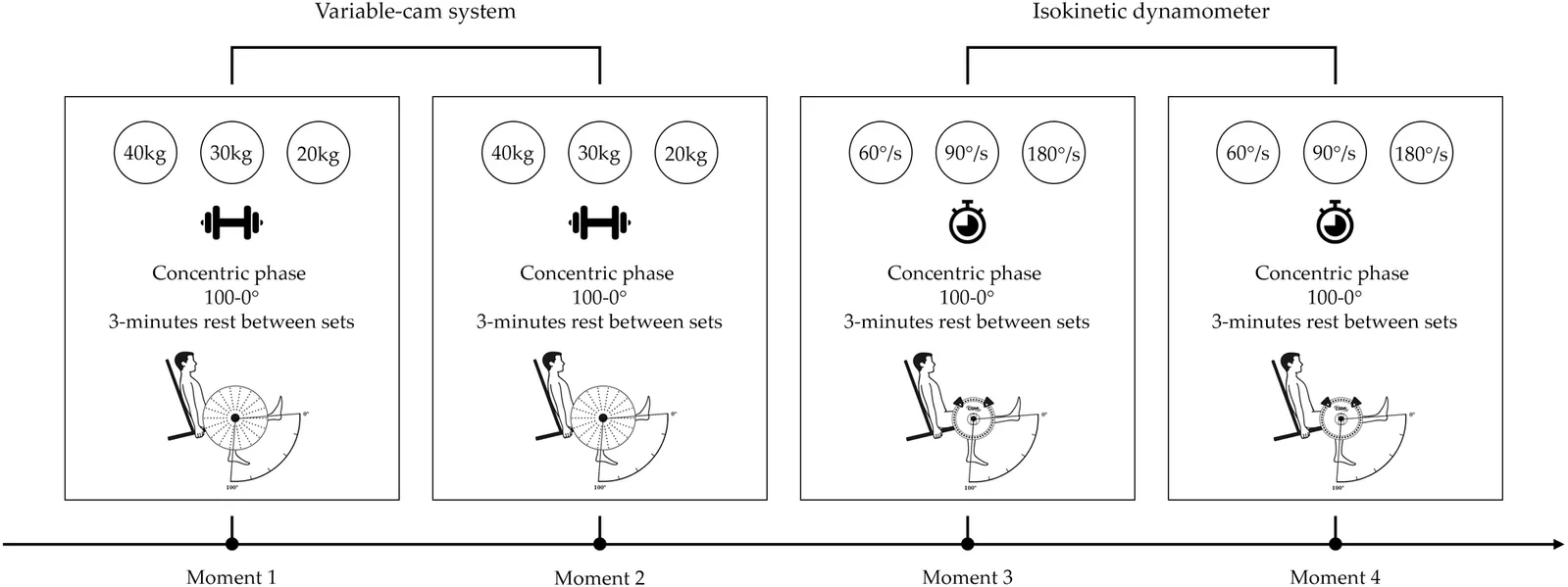
Schematic representation of the data collection protocol during leg extension for determination of torque and muscle activity during concentric contractions on the isokinetic dynamometer (Humac Norm) and on the VMAC® across the ROM (100°–0°) on both the NDS and DS.

### 2.4 Angle-torque relationship

#### 2.4.1 Variable-cam system

The VMAC®'s pin positions were tailored to match the HTC-specific strength-curve profile. Participants were positioned upright with the backrest set to 85°, and the seat was adjusted so that the knee joint aligned precisely with the rotational axis of the variable cam. Torque assessment on the VMAC® relied on a combination of a load cell and video analysis, which together enabled accurate determination of both the applied force and the corresponding moment arm during each trial (9, 10). An acrylic protractor was mounted beside the mechanism to assist in monitoring cable length and confirming the tested joint angles throughout the ROM.

Torque and displacement data were collected using a Chronojump system (Chronojump Boscosystem, Barcelona, Spain), comprising an isoinertial dynamometer equipped with a linear position transducer connected to a computer running Chronojump software (version 2.3.0). Signals were sampled at 100 Hz and processed with a 15 Hz Butterworth filter to reduce noise. The load cell was vertically attached between the leg extension base and the cable via two carabiners. A camera was placed perpendicular to the external face of the variable cam, positioned 1,500 mm away and aligned with the rotational axis, to record the moment arm length throughout the entire duration of each set. All torque values were normalized to the highest value obtained in each set.

#### 2.4.2 Isokinetic dynamometer

The Humac NORM isokinetic dynamometer was calibrated before data collection in accordance with the manufacturer's guidelines (24). The participants were seated upright with the backrest positioned at 85°, and the dynamometer was adjusted so that the knee joint aligned precisely with the device's axis of rotation. Full knee extension was defined as 0°. The lever arm pad was secured proximally to the medial malleolus to ensure that ankle movement remained unrestricted during force application. Torque signals were sampled at 100 Hz and processed using a low-pass Butterworth filter with a 15 Hz cutoff to reduce high-frequency noise. All torque values were subsequently normalized to the highest value obtained in each set.

#### 2.4.3 Surface electromyography

Data collection and reporting adhered to the sEMG standardization recommendations of Merletti (25). Surface EMG was recorded at 2,000 Hz from the rectus femoris muscle during all conditions. Bipolar electrodes (Kendall™, ECG electrodes, 57 mm in width, 34 mm in length, gel area of 201 mm^2^, sensor area of 80 mm^2^; Dublin, OH, USA) were positioned parallel to the muscle fiber orientation following the Surface Electromyography for the Non-invasive Assessment of Muscles project guidelines for electrode placement (26). For the rectus femoris muscle, electrodes were placed at 50% of the distance between the anterior superior iliac spine and the superior border of the patella. Each participant’s skin was shaved, lightly abraded, and cleaned with alcohol to ensure interelectrode impedance remained below 5 kΩ (27). A ground electrode was placed over the seventh cervical vertebra, and all electrodes were secured with double-sided adhesive tape.

Before testing, participants performed MVC trials on both their NDS and DS, following one of the most widely used normalization procedures for sEMG (28). After a standardized warm-up, each MVC trial required participants to progressively increase force and then contract “as hard and as fast as possible,” reaching maximal effort within 3–5 s, maintaining it for 3 s, and then relaxing over 3 s. At least two MVC attempts were performed, separated by 30–60 s of rest. The MVCs were executed against the static resistance of the VMAC®.

Raw sEMG signals were sampled at 2,000 Hz in differential mode, amplified, and exported as synchronized multichannel text files. Each recording was visually inspected, and a baseline segment was manually selected to compute the mean amplitude, which was subtracted from the entire signal to remove the DC offset. A fourth-order Butterworth band-pass filter (20–450 Hz) was applied to reduce motion artifacts and high-frequency noise while preserving phase characteristics. When necessary, a 60-Hz notch filter and harmonics were used to suppress power line interference. Signals were then full-wave rectified, and a 100-ms moving average filter was applied to obtain a smoothed activation envelope.

Torque was synchronized with sEMG via video, allowing for the start and end of each contraction to be identified directly from the torque–video alignment. This approach ensured precise temporal matching between muscle activation and the mechanical phases of the task, enabling joint angle measurements and signal segmentation to be made with high accuracy.

For intersubject comparisons, all sEMG data were normalized to each participant's MVC using the root mean square value as the reference. Experimental trials were segmented into 150-ms windows with 50% overlap, and the root mean square was extracted as the sole time domain feature. All processed parameters were exported as CSV files with time-aligned segmentation and visualized by automatically generated plots illustrating filtering, rectification, onset/offset detection, and envelope tracking. The entire digital pipeline was implemented in MATLAB R2015a (MathWorks, Natick, MA, USA).

### 2.5 Statistical analyses

The mean and standard deviation (SD) were calculated to characterize the distribution of the measurements. To evaluate the consistency of repeated measurements obtained with the same equipment, the CV was computed, providing a relative index of intra-device variability, defined as ideal with values below 5%, indicating excellent reproducibility, whereas values up to 15% were considered acceptable (29). The absolute reliability was estimated by calculating the standard error of measurement (SEM) and the minimum detectable change (MDC). The SEM was calculated as the standard deviation of the differences divided by the square root of 2 and the MDC was calculated as 1.96 times the square root of two times the SEM. In addition, the Bland–Altman method, which plots the difference between measurements against their mean, was used to quantify the magnitude of disagreement between the devices (30). This method also included the limit of agreement (LOA), representing the mean difference between the tests and their 95% confidence interval.

Normality was assessed using the Shapiro–Wilk test. A repeated-measures ANOVA was applied when normality was satisfied, whereas the Friedman test was used when normality was violated. For the ANOVA models, sphericity was evaluated using Mauchly's test, and the Greenhouse–Geisser correction was applied when the assumption was not met. Holm–Bonferroni corrections were applied to all multiple comparisons to control the familywise error rate.

## 3 Results

### 3.1 Torque

#### 3.1.1 Torque profiles when using the VMAC® system

The absolute torque outputs across knee joint angles in the VMAC® system under different load conditions (20, 30, and 40 kg) are presented in Figure 3 for both the NDS and DS, respectively. Both sides presented an inverted U-shaped strength curve peaking at 70°–65°.

**Figure 3:**
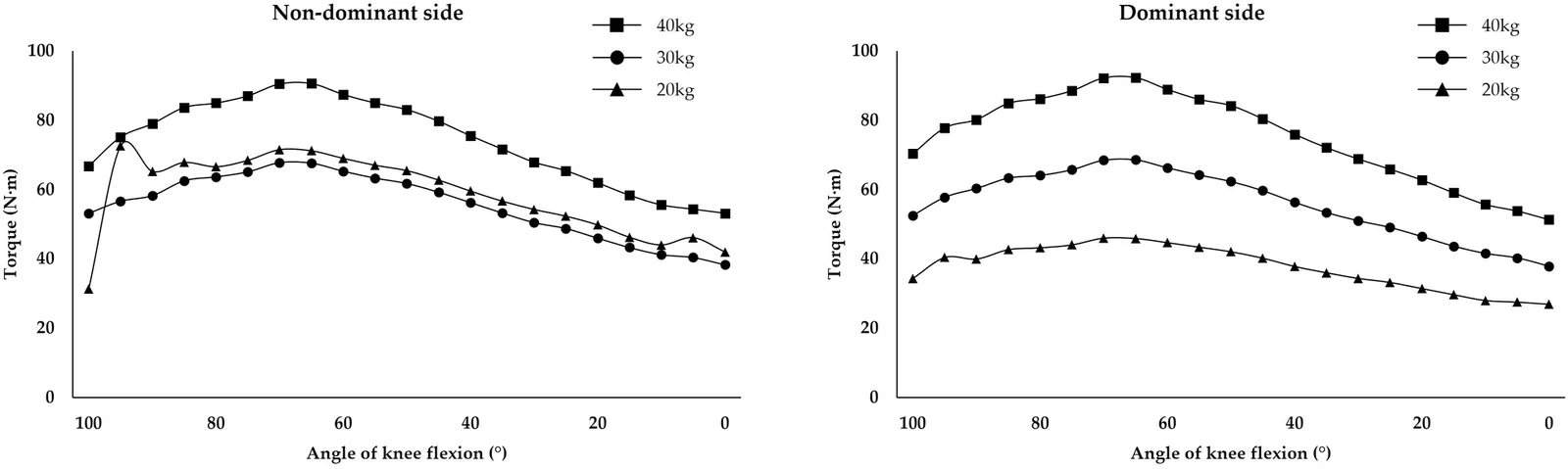
Characterization of the NDS and DS absolute torque outputs across knee joint angles on the VMAC®. 
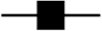
 represents the torque assessed using 40 kg, 
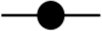
 represents the torque assessed using 30 kg, and 
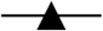
 represents the torque assessed using 20 kg.

As shown in Table 1, the torque values revealed a consistent pattern across all load conditions (40, 30, and 20 kg), increasing from the most flexed knee joint angle (100°) toward mid-ROM joint angles (70°–65°), and then decreasing until reaching full knee extension at 0°. The same overall curve shape across all loads and sides indicates that the VMAC® system produces a stable angle–torque relationship that is independent of the external load being used. This is further supported by the fact that no statistically significant differences were found among the load conditions across joint angles and sides (*p* > 0.05).

**Table 1:** Descriptive statistics, variability, and comparison between conditions (40, 30, and 20 kg) of torque values across knee joint angles on both sides on the VMAC®.

Side	Joint angle (°)	40 kg		30 kg		20 kg		Relationship between conditions	
		Mean ± SD	CV	Mean ± SD	CV	Mean ± SD	CV	Test	*p*-Value
		(% PT)	(%)	(% PT)	(%)	(% PT)	(%)		
NDS	100	75.53 ± 3.78	2.67	76.76 ± 4.83	3.41	71.57 ± 10.00	7.07	0.37^a^	0.14
	95	83.50 ± 2.89	2.04	82.59 ± 4.99	3.53	81.09 ± 4.62	3.27	0.75^a^	0.69
	90	86.46 ± 4.71	3.33	86.45 ± 3.82	2.70	85.65 ± 2.69	1.90	4.30^a^	0.12
	85	91.73 ± 4.42	3.12	91.89 ± 2.47	1.75	90.90 ± 2.42	1.71	1.91^a^	0.28
	80	93.12 ± 3.27	2.31	93.50 ± 1.89	1.34	92.61 ± 2.34	1.66	1.18^b^	0.20
	75	95.39 ± 2.39	1.69	95.60 ± 1.57	1.11	95.10 ± 1.83	1.30	2.55^a^	0.09
	70	99.14 ± 1.01	0.71	99.50 ± 0.72	0.51	99.39 ± 1.07	0.76	0.09^a^	0.16
	65	99.25 ± 1.03	0.73	99.30 ± 0.65	0.46	98.89 ± 1.39	0.98	4.21^a^	0.09
	60	95.79 ± 1.52	1.08	95.86 ± 1.14	0.80	95.77 ± 1.39	0.98	5.55^b^	0.97
	55	93.11 ± 2.33	1.65	92.98 ± 1.69	1.19	93.01 ± 1.87	1.32	15.91^b^	0.96
	50	91.02 ± 2.62	1.85	90.72 ± 1.83	1.30	90.87 ± 2.57	1.82	9.91^a^	0.28
	45	87.32 ± 2.29	1.62	87.03 ± 2.07	1.46	87.00 ± 2.54	1.80	12.09^a^	0.19
	40	82.85 ± 2.07	1.47	82.66 ± 2.20	1.55	82.62 ± 2.78	1.96	8.82^a^	0.24
	35	78.65 ± 2.15	1.52	78.31 ± 2.37	1.68	78.64 ± 3.05	2.16	0.64^a^	0.73
	30	74.50 ± 1.78	1.26	74.24 ± 2.67	1.89	75.17 ± 3.80	2.69	7.18^a^	0.55
	25	71.73 ± 1.93	1.37	71.73 ± 2.91	2.06	72.47 ± 3.73	2.64	2.38^a^	0.30
	20	68.00 ± 2.01	1.42	67.75 ± 2.46	1.74	69.02 ± 3.80	2.69	0.46^a^	0.80
	15	64.06 ± 1.80	1.27	63.84 ± 2.56	1.81	65.28 ± 3.82	2.70	0.10^a^	0.95
	10	60.53 ± 1.64	1.16	59.84 ± 1.98	1.40	62.16 ± 4.04	2.85	10.30^a^	0.61
	5	59.39 ± 2.34	1.66	58.47 ± 2.52	1.78	60.36 ± 4.02	2.84	6.78^a^	0.87
	0	57.17 ± 4.70	3.32	56.14 ± 3.00	2.12	59.17 ± 4.70	3.32	8.91^a^	0.78
DS	100	75.13 ± 2.52	1.78	73.82 ± 2.60	1.84	74.00 ± 2.11	1.49	4.67^a^	0.10
	95	82.93 ± 3.25	2.30	82.54 ± 2.50	1.77	82.45 ± 2.62	1.85	0.18^a^	0.91
	90	86.22 ± 3.46	2.44	86.23 ± 2.51	1.77	85.63 ± 2.27	1.60	1.60^a^	0.45
	85	91.36 ± 3.30	2.33	91.85 ± 1.87	1.32	91.40 ± 2.13	1.51	0.67^b^	0.51
	80	92.81 ± 3.17	2.24	92.98 ± 1.55	1.09	92.86 ± 1.43	1.01	0.64^a^	0.73
	75	95.23 ± 2.61	1.84	95.35 ± 1.46	1.03	95.02 ± 2.50	1.76	0.64^a^	0.73
	70	99.17 ± 1.42	1.00	99.35 ± 0.79	0.56	99.17 ± 2.22	1.57	1.76^a^	0.41
	65	99.27 ± 0.83	0.59	99.48 ± 0.59	0.42	98.86 ± 1.97	1.39	1.54^a^	0.14
	60	95.64 ± 1.57	1.11	96.10 ± 1.03	0.73	95.93 ± 0.86	0.61	1.08^b^	0.34
	55	92.56 ± 2.05	1.45	93.15 ± 1.39	0.98	92.97 ± 1.39	0.98	1.73^a^	0.42
	50	90.62 ± 2.23	1.58	90.42 ± 1.98	1.40	90.46 ± 2.42	1.71	0.15^b^	0.86
	45	86.91 ± 2.61	1.84	86.56 ± 2.24	1.59	86.67 ± 3.41	2.41	2.82^a^	0.24
	40	82.14 ± 3.01	2.13	81.71 ± 2.28	1.61	81.70 ± 3.76	2.66	2.09^a^	0.35
	35	78.00 ± 2.71	1.92	77.48 ± 2.25	1.59	77.71 ± 3.55	2.51	1.18^a^	0.55
	30	74.51 ± 2.43	1.72	74.00 ± 1.88	1.33	74.11 ± 2.63	1.86	1.46^a^	0.48
	25	71.35 ± 2.00	1.42	71.23 ± 1.72	1.21	71.59 ± 2.50	1.76	0.35^b^	0.64
	20	67.27 ± 2.13	1.51	67.44 ± 1.83	1.29	67.78 ± 2.35	1.66	2.50^a^	0.29
	15	63.34 ± 1.86	1.32	63.29 ± 1.61	1.14	64.08 ± 2.76	1.95	4.46^a^	0.95
	10	59.90 ± 0.84	0.59	60.14 ± 1.47	1.04	60.63 ± 2.60	1.84	0.73^a^	0.70
	5	57.67 ± 1.38	0.97	58.24 ± 1.53	1.08	59.32 ± 2.20	1.56	3.82^a^	0.15
	0	55.46 ± 2.01	1.42	55.76 ± 1.94	1.37	58.11 ± 2.87	2.03	3.25^a^	0.07

NDS, non-dominant side; DS, dominant side; SD, standard deviation; CV, coefficient of variation; PT, peak torque.

a Friedman’s test.

b Repeated-measures ANOVA.

On the NDS, the highest torque, 99.25 ± 1.03% PT, was reached at 65° under the 40 kg condition. Similarly, under the 30 and 20 kg conditions, the highest torque values were 99.50 ± 0.72% PT and 99.39 ± 1.07% PT, respectively, and both were reached at 70°. For the DS, the highest torque values were 99.27 ± 0.83% PT at 65°, 99.48 ± 0.59% PT at 65°, and 99.17 ± 2.22% PT at 70°, under the 40, 30, and 20 kg conditions, respectively. These findings suggest that the mid-ROM region of the movement offers a consistent mechanical advantage.

In addition, the CVs obtained for the torque measurements (0.71%–7.07% on the NDS and 0.42%–2.66% on the DS) were, in general, below the 5% cutoff for excellent reproducibility and always within the acceptable range of 15%, which demonstrates the internal consistency of the VMAC® system.

#### 3.1.2 Torque profiles when using the isokinetic dynamometer

The absolute torque outputs across knee joint angles on the isokinetic dynamometer at different angular velocities (60, 90, and 180°/s) are presented in Figure 4 for both the NDS and DS, respectively. Both sides presented an inverted U-shaped strength curve peaking at 65°–60°, with the DS recording higher values.

**Figure 4:**
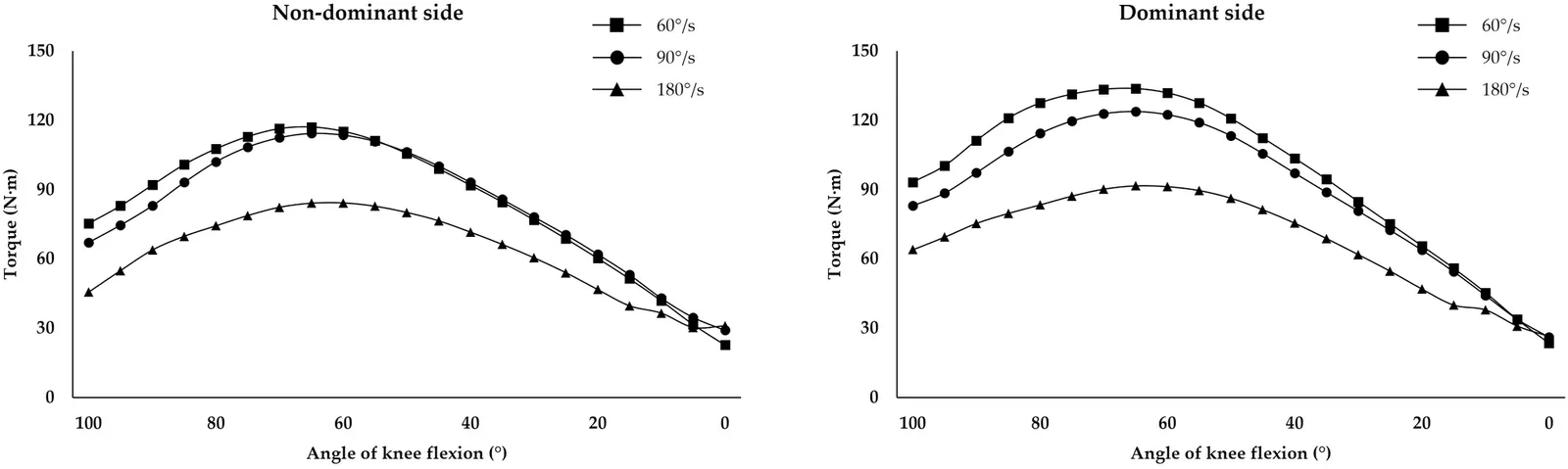
Characterization of the NDS and DS absolute torque outputs across knee joint angles on the isokinetic dynamometer. 
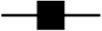
 represents the torque assessed at 60°/s, 
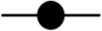
 represents the torque assessed at 90°/s, and 
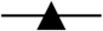
 represents the torque assessed at 180°/s.

As shown in Table 2, the torque values for both the NDS and DS exhibit velocity-dependent angle–torque curves, with higher angular velocities being associated with lower torque values across the ROM. After Holm–Bonferroni correction, statistically significant differences between torque values at different velocities were found at 55° and 45° on the NDS. However, on the DS, there were no statistically significant differences observed, which suggests that there is a greater torque consistency across angular velocities on the DS. Similar to the VMAC® system, the torque values obtained using the isokinetic dynamometer followed the pattern of progressively increasing from the initial angle of movement (100°) toward the mid-ROM joint angles, reaching a peak around 65°, and subsequently decreasing until reaching full extension (0°).

**Table 2:** Descriptive statistics, variability, and comparison between conditions (60, 90, and 180°/s) of torque values across knee joint angles for both sides in the isokinetic dynamometer.

Side	Joint angle (°)	60°/s		90°/s		180°/s		Relationship between conditions	
		Mean ± SD	CV	Mean ± SD	CV	Mean ± SD	CV	Test	*p-*Value
		(% PT)	(%)	(% PT)	(%)	(% PT)	(%)		
NDS	100	58.88 ± 23.97	40.70	55.87 ± 24.12	43.18	54.22 ± 24.80	45.74	0.37^a^	0.83
	95	65.96 ± 18.80	28.50	62.52 ± 20.59	32.94	60.95 ± 22.26	36.52	1.03^b^	0.37
	90	71.73 ± 15.81	22.05	68.86 ± 16.29	23.66	69.53 ± 18.41	26.48	0.48^b^	0.62
	85	78.07 ± 15.04	19.26	76.94 ± 12.70	16.50	76.23 ± 14.95	19.61	1.91^a^	0.39
	80	83.33 ± 14.33	17.20	84.30 ± 9.98	11.84	82.16 ± 11.39	13.86	1.18^a^	0.55
	75	87.54 ± 13.29	15.18	89.57 ± 8.78	9.80	87.68 ± 7.67	8.75	2.55^a^	0.28
	70	90.30 ± 12.15	13.46	92.99 ± 8.74	9.40	91.93 ± 5.62	6.11	0.09^a^	0.96
	65	91.01 ± 11.45	12.58	94.73 ± 8.80	9.29	94.14 ± 5.42	5.75	4.21^a^	0.12
	60	89.94 ± 10.94	12.16	94.33 ± 8.69	9.21	94.45 ± 6.04	6.40	5.55^a^	0.06
	55	87.39 ± 10.55	12.07	92.43 ± 8.34	9.03	92.83 ± 7.27	7.83	15.91^a^	<0.001**
	50	83.57 ± 10.34	12.37	88.92 ± 7.89	8.87	89.80 ± 8.11	9.03	9.91^a^	0.007*
	45	79.03 ± 10.67	13.51	84.30 ± 7.51	8.91	85.74 ± 8.23	9.60	12.09^a^	0.002**
	40	73.96 ± 11.49	15.54	78.65 ± 7.62	9.69	80.42 ± 8.38	10.42	8.82^a^	0.01*
	35	68.56 ± 12.68	18.49	72.65 ± 7.91	10.89	74.55 ± 8.19	10.99	3.80^b^	0.46
	30	62.67 ± 13.34	21.29	66.39 ± 8.51	12.83	68.20 ± 8.01	11.74	7.18^a^	0.03*
	25	56.41 ± 13.11	23.25	60.12 ± 9.02	15.00	60.99 ± 8.20	13.45	2.24^b^	0.13
	20	49.65 ± 12.63	25.44	53.30 ± 9.38	17.59	52.87 ± 8.98	16.99	1.66^b^	0.21
	15	42.68 ± 12.18	28.54	45.97 ± 9.16	19.93	44.87 ± 9.41	20.98	1.39^b^	0.26
	10	35.08 ± 11.77	33.55	37.49 ± 8.99	23.97	39.54 ± 10.57	26.72	10.30^a^	0.01*
	5	26.35 ± 10.62	40.32	29.75 ± 9.31	31.30	30.86 ± 11.29	36.57	6.78^a^	0.03*
	0	19.24 ± 10.21	53.08	23.96 ± 9.49	39.62	30.30 ± 9.35	30.87	8.91^a^	0.01*
DS	100	65.65 ± 25.54	38.91	64.20 ± 28.38	44.21	65.15 ± 27.15	41.67	0.11^b^	0.09
	95	69.56 ± 19.65	28.25	68.12 ± 21.59	31.70	69.98 ± 21.74	31.07	0.29^b^	0.70
	90	76.33 ± 15.92	20.85	74.07 ± 16.00	21.60	76.08 ± 16.30	21.42	0.59^b^	0.51
	85	82.58 ± 13.73	16.63	80.74 ± 12.68	15.71	80.16 ± 12.82	15.99	0.57^b^	0.50
	80	86.98 ± 12.17	13.99	86.65 ± 10.05	11.60	83.90 ± 10.18	12.14	1.03^b^	0.34
	75	89.80 ± 10.33	11.50	90.87 ± 8.37	9.21	87.91 ± 9.21	10.47	0.90^b^	0.37
	70	91.65 ± 9.17	10.00	93.56 ± 7.15	7.64	91.21 ± 8.73	9.57	0.75^b^	0.43
	65	92.40 ± 9.00	9.74	94.49 ± 7.00	7.41	92.78 ± 9.24	9.96	1.54^a^	0.46
	60	91.57 ± 9.93	10.85	93.68 ± 7.43	7.93	92.61 ± 9.78	10.56	0.71^b^	0.78
	55	88.93 ± 11.05	12.43	91.40 ± 8.29	9.06	91.11 ± 10.13	11.12	1.13^b^	0.30
	50	84.27 ± 11.60	13.77	87.42 ± 9.36	10.71	87.87 ± 10.84	12.33	2.41^b^	0.12
	45	78.43 ± 11.21	14.29	81.90 ± 10.04	12.26	82.98 ± 11.40	13.73	3.07^b^	0.07
	40	72.46 ± 10.76	14.85	75.72 ± 10.07	13.30	77.49 ± 12.01	15.50	2.90^b^	0.08
	35	66.30 ± 10.33	15.59	69.45 ± 9.78	14.08	70.97 ± 12.68	17.87	2.09^b^	0.15
	30	59.60 ± 9.78	16.41	63.18 ± 9.13	14.45	63.90 ± 13.13	20.55	1.69^b^	0.21
	25	52.94 ± 8.94	16.89	56.68 ± 8.14	14.36	56.42 ± 12.91	22.88	1.34^b^	0.27
	20	46.25 ± 7.98	17.26	50.03 ± 7.18	14.35	48.38 ± 12.74	26.34	1.11^b^	0.32
	15	39.43 ± 6.98	17.71	42.79 ± 6.34	14.82	41.08 ± 11.94	29.07	4.46	0.11
	10	31.99 ± 6.32	19.77	34.76 ± 6.15	17.68	38.08 ± 13.72	36.03	2.00^b^	0.18
	5	23.74 ± 6.20	26.11	26.74 ± 6.51	24.35	32.44 ± 15.56	47.97	3.44^b^	0.08
	0	16.47 ± 6.10	37.06	21.95 ± 6.77	30.82	23.17 ± 8.65	37.34	3.25^a^	0.20

NDS, non-dominant side; DS, dominant side; SD, standard deviation; CV, coefficient of variation; PT, peak torque.

a Friedman’s test.

b Repeated-measures ANOVA.

* *p* ≤ 0.05 (uncorrected).

** *p* ≤ 0.05 after Holm–Bonferroni correction.

On the NDS, the highest torque values were 91.01 ± 11.45% PT and 94.73 ± 8.80% PT for 60°/s and 90°/s, respectively, and both were reached at 65°. At 180°/s, the highest torque value was 94.45 ± 6.04% PT and was reached at 60°. On the DS, the highest torque values were 92.40 ± 9.00% PT, 94.49 ± 7.00% PT, and 92.78 ± 9.24% PT, all at 65°, under the 60°/s, 90°/s, and 180°/s conditions, respectively. Once more, these findings corroborate that there is a mechanical advantage at the mid-ROM joint angles.

The CV values computed for the dynamometer torque measurements were substantially high, reaching maximum values of 53.08% at 0° on the NDS and 47.97% at 5° on the DS, with a significant amount of the measurements not even within the acceptable range (< 15%). The elevated CV values were most pronounced at the extreme joint angles, i.e., when reaching maximum flexion or full extension, and less severe around the mid-ROM joint angles, where acceptable levels of variability were reached.

#### 3.1.3 Between-equipment relationship

Considering that the VMAC® had no significant differences between conditions on the NDS (*p* > 0.05), the values presented in Table 3 are the mean values recorded under different conditions. Therefore, the presented between-equipment relationship compares the values obtained under each condition on the isokinetic dynamometer with the mean values from the VMAC®.

**Table 3:** Descriptive statistics, variability, and between-equipment comparison of torque values across knee joint angles on the NDS.

Joint angle (°)	Isokinetic dynamometer			VMAC®		Between-equipment relationship					
	60°/s	90°/s	180°/s	Mean values		60°/s vs. VMAC®		90°/s vs. VMAC®		180°/s vs. VMAC®	
	Mean ± SD	Mean ± SD	Mean ± SD	Mean ± SD	CV	SEM	MDC	SEM	MDC	SEM	MDC
	(% PT)	(% PT)	(% PT)	(% PT)	(%)	(% PT)	(% PT)	(% PT)	(% PT)	(% PT)	(% PT)
100	58.88 ± 23.97	55.87 ± 24.12	54.22 ± 24.80	76.44 ± 5.03	6.58	18.73	51.92	19.25	53.36	20.49	56.80
95	65.96 ± 18.80	62.52 ± 20.59	60.95 ± 22.26	82.41 ± 3.81	4.62	14.21	39.39	15.77	43.71	14.03	38.89
90	71.73 ± 15.81	68.86 ± 16.29	69.53 ± 18.41	86.21 ± 3.32	3.85	9.08	25.17	11.61	32.18	11.94	33.10
85	78.07 ± 15.04	76.94 ± 12.70	76.23 ± 14.95	91.51 ± 2.66	2.90	9.58	26.55	9.19	25.47	10.30	28.55
80	83.33 ± 14.33	84.30 ± 9.98	82.16 ± 11.39	93.08 ± 2.21	2.37	9.06	25.11	6.93	19.21	7.77	21.54
75	87.54 ± 13.29	89.57 ± 8.78	87.68 ± 7.67	95.37 ± 1.57	1.64	8.42	23.34	6.10	16.91	5.10	14.14
70	90.30 ± 12.15	92.99 ± 8.74	91.93 ± 5.62	99.34 ± 0.79	0.80	8.55	23.70	6.19	17.16	4.02	11.14
65	91.01 ± 11.45	94.73 ± 8.80	94.14 ± 5.42	99.15 ± 0.72	0.73	7.97	22.09	6.03	16.71	3.64	10.09
60	89.94 ± 10.94	94.33 ± 8.69	94.45 ± 6.04	95.81 ± 0.93	0.97	6.87	19.04	5.10	14.14	3.23	8.95
55	87.39 ± 10.55	92.43 ± 8.34	92.83 ± 7.27	93.03 ± 1.46	1.57	6.40	17.74	4.30	11.92	2.99	8.29
50	83.57 ± 10.34	88.92 ± 7.89	89.80 ± 8.11	90.87 ± 1.59	1.75	5.90	16.35	3.68	10.20	3.54	9.81
45	79.03 ± 10.67	84.30 ± 7.51	85.74 ± 8.23	87.12 ± 1.65	1.89	5.42	15.02	3.36	9.31	3.79	10.51
40	73.96 ± 11.49	78.65 ± 7.62	80.42 ± 8.38	82.71 ± 1.51	1.83	5.35	14.83	3.35	9.29	4.29	11.89
35	68.56 ± 12.68	72.65 ± 7.91	74.55 ± 8.19	78.53 ± 1.54	1.96	5.64	15.63	4.22	11.70	4.36	12.09
30	62.67 ± 13.34	66.39 ± 8.51	68.20 ± 8.01	74.64 ± 1.54	2.06	5.86	16.24	4.97	13.78	4.40	12.20
25	56.41 ± 13.11	60.12 ± 9.02	60.99 ± 8.20	72.04 ± 1.88	2.62	6.87	19.04	5.68	15.74	4.29	11.89
20	49.65 ± 12.63	53.30 ± 9.38	52.87 ± 8.98	68.32 ± 1.77	2.60	8.02	22.23	6.07	16.83	4.96	13.75
15	42.68 ± 12.18	45.97 ± 9.16	44.87 ± 9.41	64.45 ± 1.66	2.57	8.41	23.31	6.59	18.27	6.36	17.63
10	35.08 ± 11.77	37.49 ± 8.99	39.54 ± 10.57	61.07 ± 1.54	2.52	8.26	22.90	6.42	17.80	10.73	29.74
5	26.35 ± 10.62	29.75 ± 9.31	30.86 ± 11.29	59.64 ± 1.98	3.32	7.29	20.21	6.73	18.65	10.61	29.41
0	19.24 ± 10.21	23.96 ± 9.49	30.30 ± 9.35	57.68 ± 2.58	4.48	9.49	26.30	11.09	30.74	12.32	34.15

SD, standard deviation; CV, coefficient of variation; SEM, standard error of measurement; MDC, minimal detectable change; PT, peak torque.

On the NDS, the VMAC® system consistently achieved higher torque values throughout the entire ROM compared with the isokinetic dynamometer, with the greatest difference observed at the full extension phase (0°), where the torque value on the VMAC® was 57.68 ± 2.58% PT, while the isokinetic dynamometer's value was 19.24 ± 10.21% PT at 60°/s. Consistently low SEM and MDC values were found across almost the entire ROM (80°–5°), indicating good reliability, except for extreme joint angles, where the SEM and MDC values were higher, reflecting higher variability and less torque stability.

Neither the VMAC® nor the isokinetic dynamometer had significant within-condition differences on the DS (*p* > 0.05); therefore, the values presented in Table 4 are mean values and the presented between-equipment relationship compares the mean values from the isokinetic dynamometer with the mean values from the VMAC®.

**Table 4:** Descriptive statistics, variability, and between-equipment comparison of torque values across knee joint angles on the DS.

Joint angle (°)	Isokinetic dynamometer		VMAC®		Between-equipment relationship	
	Mean values		Mean values			
	Mean ± SD	CV	Mean ± SD	CV	SEM	MDC
	(% PT)	(%)	(% PT)	(%)	(% PT)	(% PT)
100	65.00 ± 25.63	39.43	74.60 ± 2.22	2.97	22.44	62.20
95	69.22 ± 19.83	28.66	82.69 ± 2.20	2.66	19.66	54.49
90	75.49 ± 14.83	19.64	86.05 ± 2.46	2.86	9.37	25.97
85	81.16 ± 11.39	14.03	91.54 ± 2.17	2.37	7.44	20.62
80	85.84 ± 8.75	10.2	92.88 ± 1.82	1.96	5.20	14.41
75	89.53 ± 7.09	7.92	95.20 ± 1.48	1.56	4.13	11.45
70	92.14 ± 6.32	6.85	99.23 ± 1.14	1.15	4.30	11.92
65	93.22 ± 6.73	7.22	99.20 ± 0.69	0.70	4.74	13.14
60	92.62 ± 7.77	8.39	95.89 ± 0.84	0.87	4.57	12.67
55	90.48 ± 8.73	9.65	92.90 ± 1.29	1.38	4.73	13.11
50	86.52 ± 9.48	10.95	90.50 ± 1.95	2.15	5.16	14.30
45	81.10 ± 9.59	11.82	86.72 ± 2.44	2.81	5.35	14.83
40	75.22 ± 9.36	12.45	81.85 ± 2.64	3.23	5.04	13.97
35	68.91 ± 9.02	13.09	77.73 ± 2.45	3.16	5.25	14.55
30	62.23 ± 8.45	13.58	74.21 ± 2.00	2.69	5.46	15.13
25	55.35 ± 7.52	13.59	71.39 ± 1.74	2.43	5.16	14.30
20	48.22 ± 6.73	13.96	67.57 ± 1.85	2.73	4.68	12.97
15	41.10 ± 6.21	15.11	63.71 ± 1.74	2.74	4.42	12.25
10	34.71 ± 5.35	15.43	60.37 ± 1.50	2.48	3.98	11.03
5	26.95 ± 5.42	20.12	58.67 ± 1.55	2.64	4.25	11.78
0	19.79 ± 4.12	20.81	56.78 ± 2.11	3.71	5.39	14.94

SD, standard deviation; CV, coefficient of variation; SEM, standard error of measurement; MDC, minimal detectable change; PT, peak torque.

On the DS, the VMAC® system consistently produced higher torque values across the entire ROM when compared with the isokinetic dynamometer. The greatest discrepancy occurred at full extension (0°), where the VMAC® maintained 56.78 ± 2.11% PT, whereas the isokinetic dynamometer dropped to 19.79 ± 4.12% PT. The SEM and MDC values were consistently low throughout the majority of the ROM (90°–0°), indicating good reliability. The only exceptions were the initial joint angles (100° and 95°), where higher SEM and MDC values reflected increased variability and reduced torque stability.

#### 3.1.4 Agreement analysis

The level of agreement between the torque values obtained with the VMAC® system and with the isokinetic dynamometer was assessed using Bland–Altman plots, which are presented in Figures 5–8. For the NDS, the Bland-Altman plots are presented using the values obtained under different conditions on the isokinetic dynamometer against the mean values obtained on the VMAC®. For the DS, the Bland–Altman plots are presented using the mean values obtained on both pieces of equipment.

**Figure 5:**
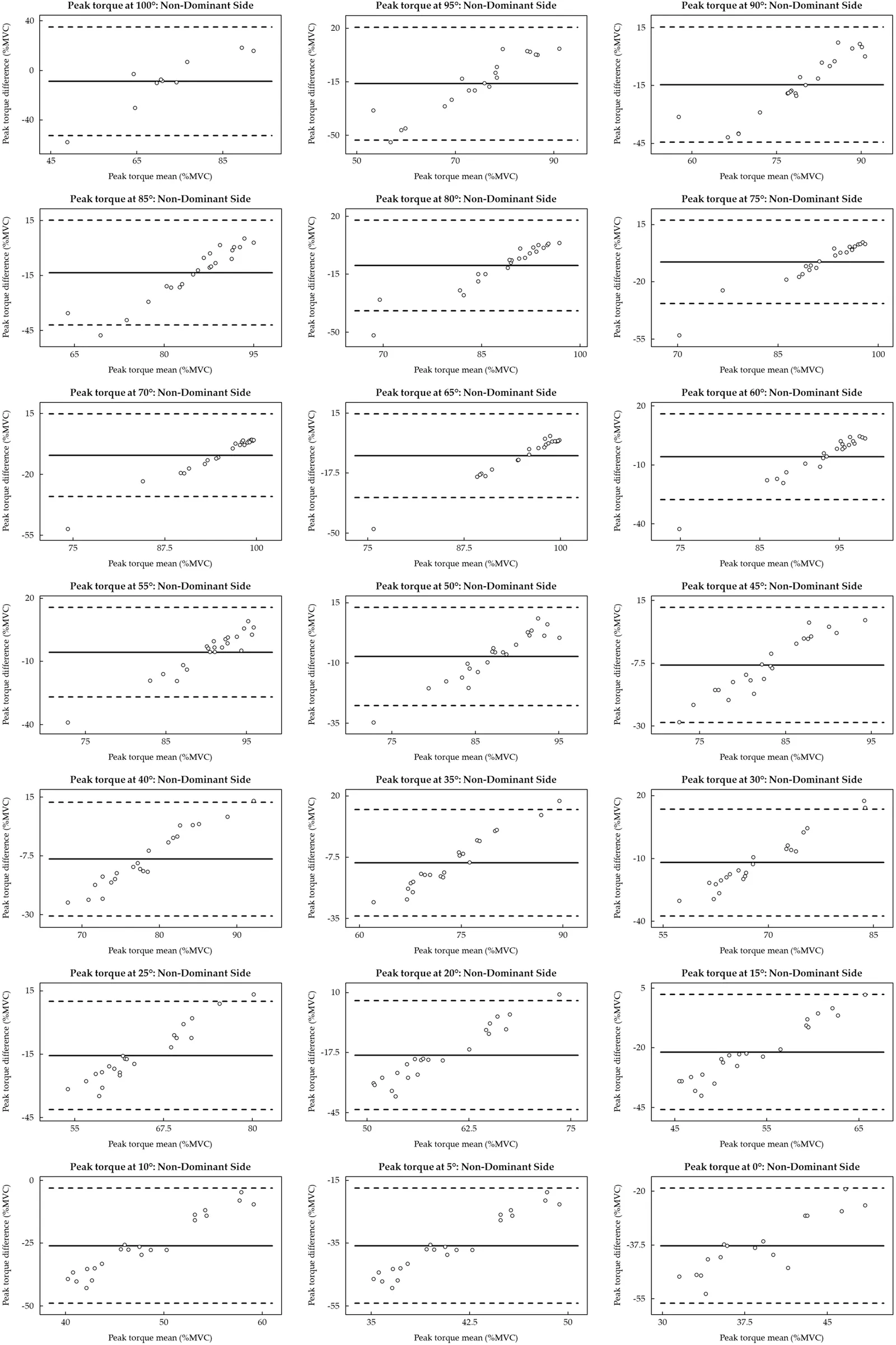
These Bland–Altman plots illustrate the agreement between the NDS torque values across knee joint angles, comparing the 60°/s isokinetic dynamometer measurements with VMAC®-derived means. Differences are shown relative to their means, with dashed lines marking the LOA (bias ± 1.96 SD).

**Figure 6:**
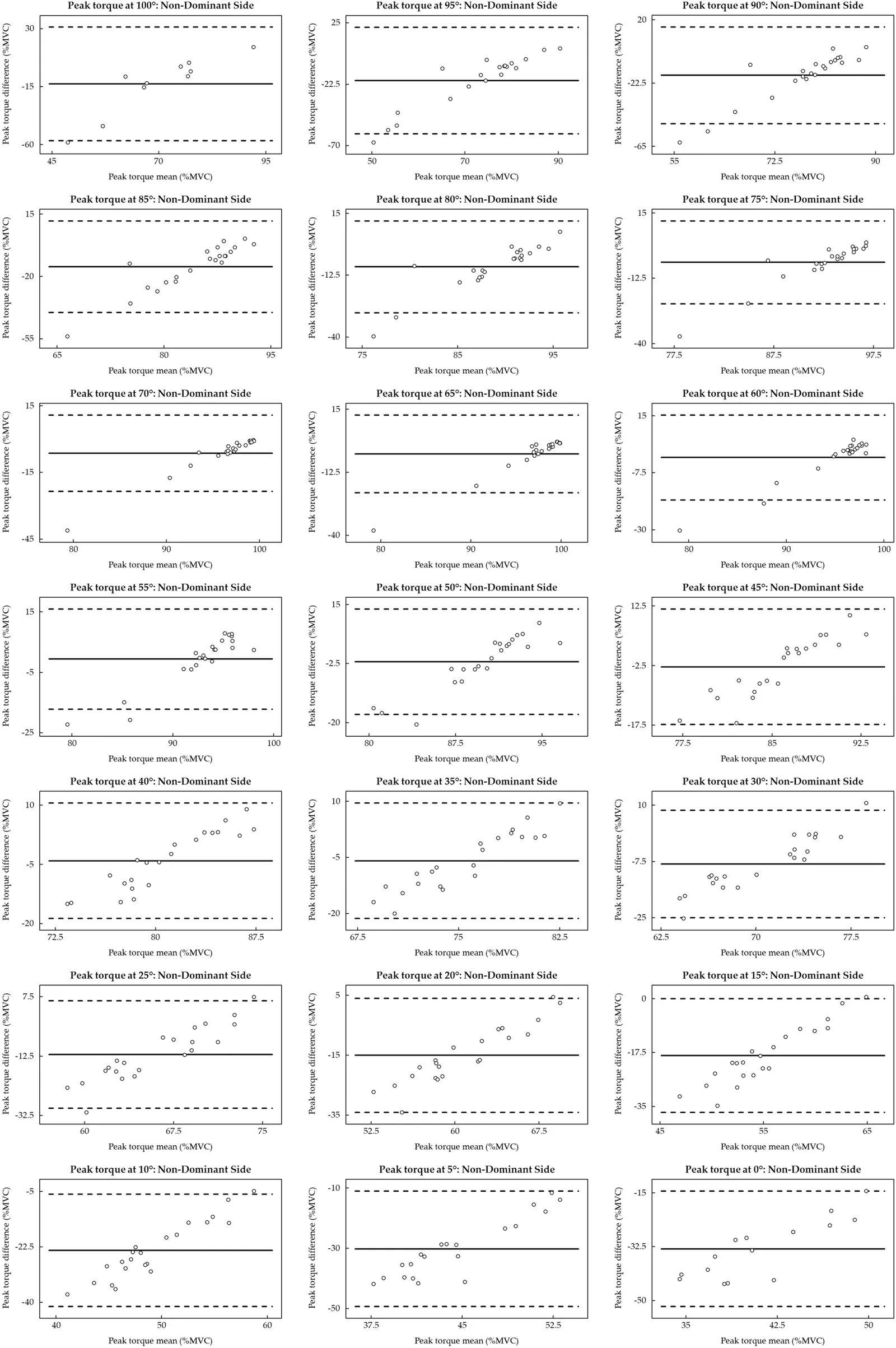
These Bland–Altman plots illustrate the agreement between the NDS torque values across knee joint angles, comparing the 90°/s isokinetic dynamometer measurements with VMAC®-derived means. Differences are shown relative to their means, with dashed lines marking the LOA (bias ± 1.96 SD).

**Figure 7:**
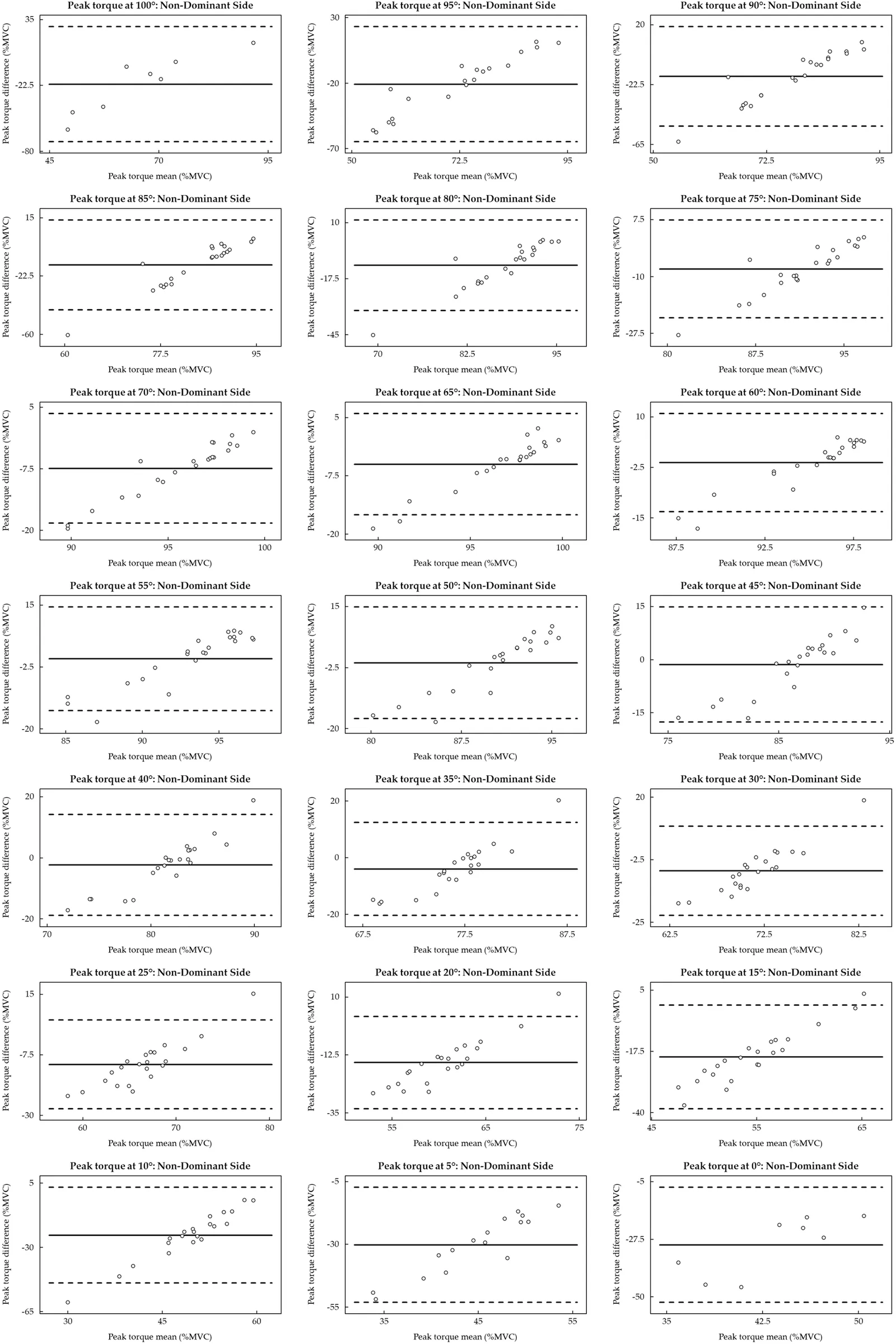
These Bland–Altman plots illustrate the agreement between the NDS torque values across knee joint angles, comparing the 180°/s isokinetic dynamometer measurements with VMAC®-derived means. Differences are shown relative to their means, with dashed lines marking the LOA (bias ± 1.96 SD).

**Figure 8:**
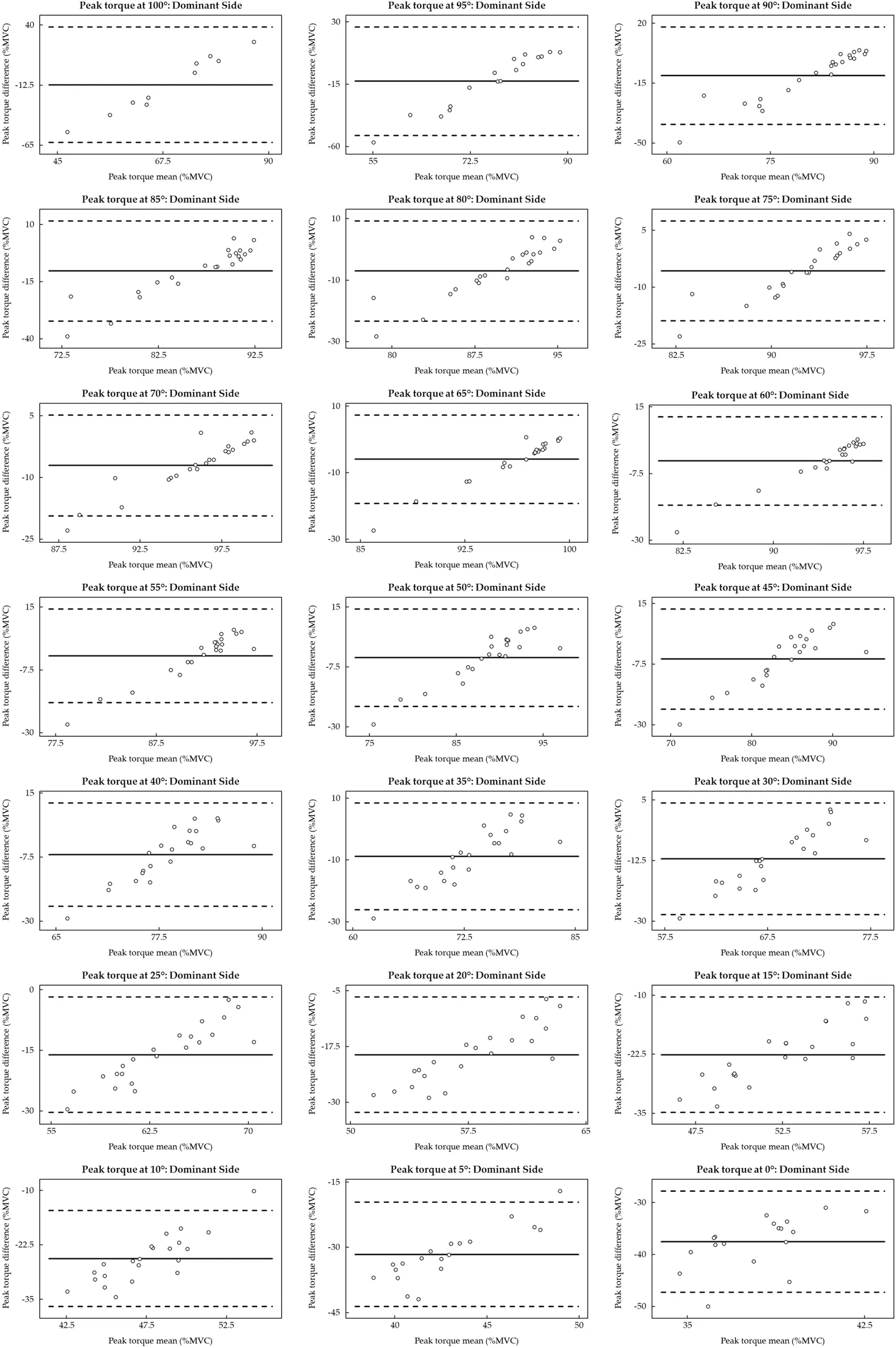
These Bland–Altman plots illustrate the agreement between the DS torque values across knee joint angles, comparing the isokinetic dynamometer- and VMAC®-derived means. Differences are shown relative to their means, with dashed lines marking the LOA (bias ± 1.96 SD).

The Bland–Altman plots comparing the 60°/s isokinetic dynamometer measurements with VMAC®-derived means on the NDS (Figure 5) showed that the bias varied between −37.73 and −5.64, with the highest value at 55° and the lowest value at 0°. Furthermore, the lower LOA varied between −56.47 and −26.86, with the highest and lowest values also at 55 and 0°, respectively, and the upper LOA varied between −18.98 and 35.23, with the highest value observed at 100° and the lowest value observed at 0°.

The Bland–Altman plots comparing the 90°/s isokinetic dynamometer measurements with VMAC®-derived means on the NDS (Figure 6) showed that the bias varied from −33.24 to −0.60, with the highest value being observed at 55° and the lowest value observed at 0°. Furthermore, the lower LOA varied between −60.85 and −17.20, with the highest value observed at 55° and the lowest value observed at 95°, and the upper LOA varied from −14.49 to 31.26, corresponding to 0° and 100°, respectively.

Comparing the 180°/s isokinetic dynamometer measurements with VMAC®-derived means on the NDS (Figure 7), the Bland–Altman plots showed that the bias varied between −30.18 and −0.20, corresponding to 0° and 55°, respectively. Furthermore, the lower LOA varied between −71.58 and −13.50, with the highest value observed at 60° and the lowest value observed at 100°, and the upper LOA ranged from −7.32 to 28.86, corresponding to 5° and 100°, respectively.

The Bland–Altman plots comparing the mean values obtained on the isokinetic dynamometer and the mean values obtained on the VMAC® on the DS (Figure 8) showed bias ranging from −37.54 to −2.42, corresponding to 0° and 55°, respectively. Furthermore, the plots showed lower LOA values varying between −62.60 and −18.21, corresponding to 100° and 60°, and upper LOA values ranging from −27.80 to 38.32, corresponding to 0° and 100°.

#### 3.1.5 Strength curves

Figure 9 presents the strength curves obtained on the NDS on both the VMAC® system and the isokinetic dynamometer. The presented strength curves correspond to the mean values obtained on the VMAC® and the values obtained under different conditions on the isokinetic dynamometer.

**Figure 9:**
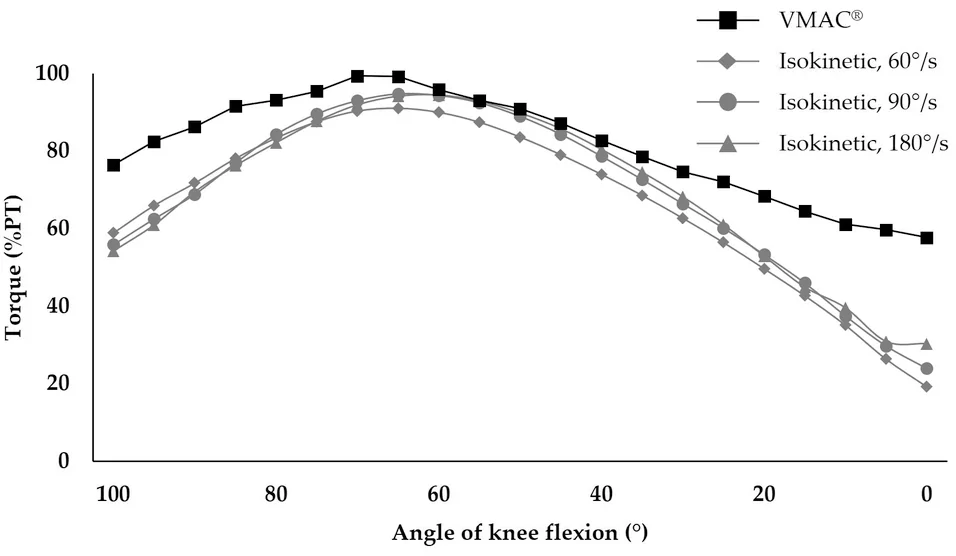
Characterization of the NDS strength curves across knee joint angles assessed on the VMAC® and isokinetic dynamometer. 
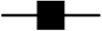
 represents the torque assessed on the VMAC®, 
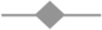
 represents the torque assessed at 60°/s on the isokinetic dynamometer, 
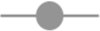
 represents the torque assessed at 90°/s on the isokinetic dynamometer, and 
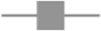
 represents the torque assessed at 180°/s on the isokinetic dynamometer.

The NDS strength curves obtained on the VMAC® system closely matched those obtained on the isokinetic dynamometer across the initial and mid-ROM joint angles (100°–35°), reaching their PT value at around the mid-ROM (approximately 70°–65°), indicating that the VMAC® system is able to successfully reproduce the characteristic angle–torque relationship in knee extension exercises at the majority of ROM. Particularly at the final-ROM joint angles (35°–0°), the VMAC® had a flatter descending phase, whereas the one produced by the isokinetic dynamometer showed a more pronounced decline in the final stages of the movement.

The strength curves obtained on the DS on both the VMAC® system and the isokinetic dynamometer are presented in Figure 10. The presented strength curves correspond to the mean values obtained on the VMAC® and on the isokinetic dynamometer.

**Figure 10:**
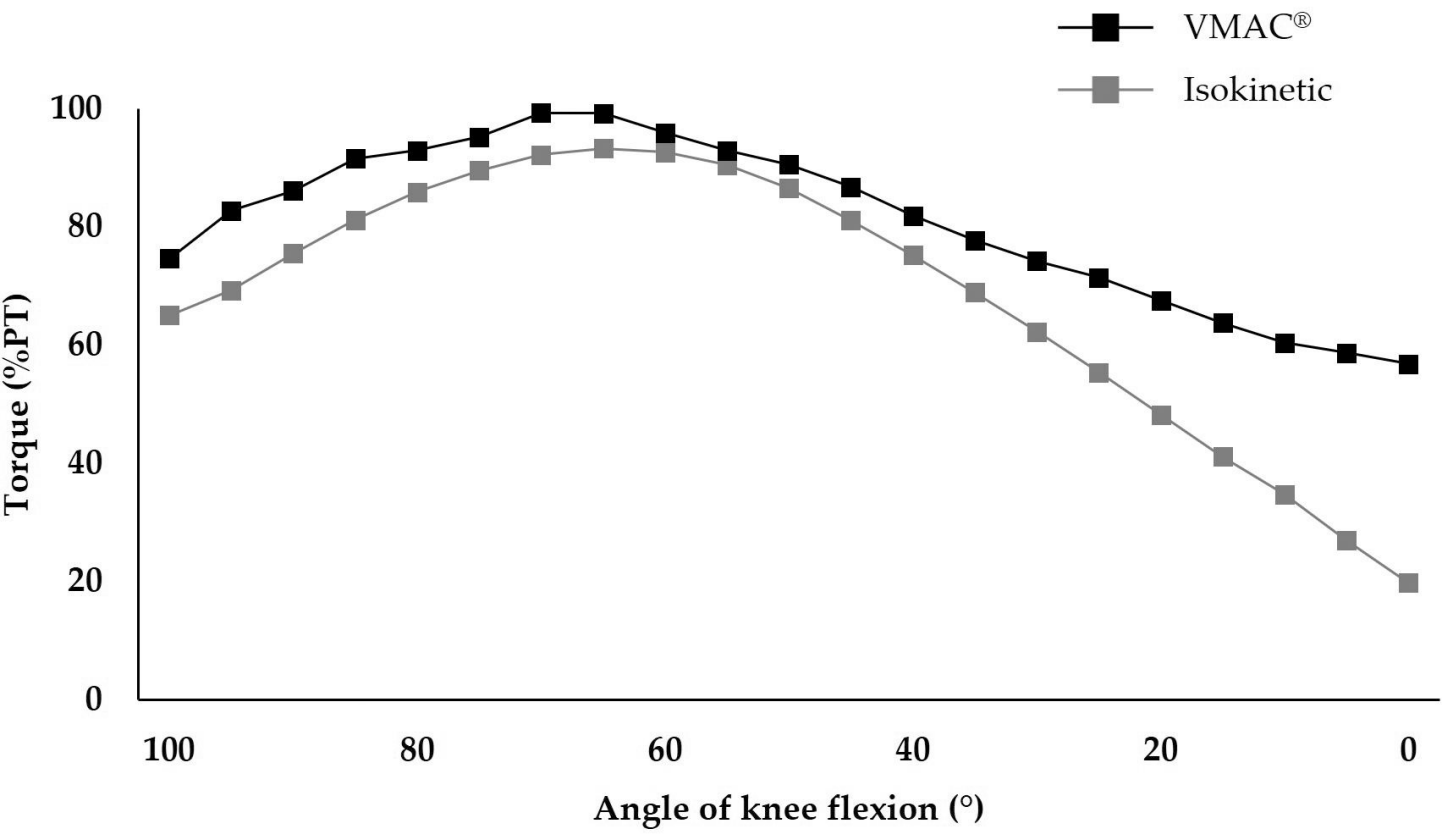
Characterization of the DS strength curves across knee joint angles assessed on the VMAC® and isokinetic dynamometer. 
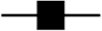
 represents the torque assessed on the VMAC® and 
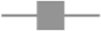
 represents the torque assessed on the isokinetic dynamometer.

Similar to the NDS, the VMAC® system produced strength curves that closely matched those observed on the isokinetic dynamometer across the initial and mid-ROM joint angles (100°–35°), peaking around mid-ROM (70°–65°). Moreover, at the final-ROM joint angles (35°–0°), the VMAC® presented a flatter descending strength curve compared to that obtained on the isokinetic dynamometer.

### 3.2 Muscle activity

Muscle activity across all load conditions (40, 30, and 20 kg) recorded on the VMAC® system on both the NDS and DS is presented in Table 5. The VMAC®'s muscle activation values increased with higher loads, reflecting a load-dependent pattern, which was further supported by the statistically significant differences found in muscle activation between conditions (*p* < 0.05) at the majority of joint angles on both sides (95°–5°). CV values remained within the acceptable range (<15%) across the entire ROM, with slightly higher variability at larger joint angles and lower loads, except at 0° with a 30 kg load (15.42%).

**Table 5:** Descriptive statistics, variability, and between-conditions comparison (40, 30, and 20 kg) of muscle activity values across knee joint angles on both sides on the VMAC®.

Side	Joint angle (°)	40 kg		30 kg		20 kg		Relationship between conditions	
		Mean ± SD	CV	Mean ± SD	CV	Mean ± SD	CV	Test	*p-*Value
		(% MVC)	(%)	(% MVC)	(%)	(% MVC)	(%)		
NDS	100	14.69 ± 14.48	10.24	11.05 ± 9.85	6.97	3.08 ± 1.81	1.28	0.01^a^	1.00
	95	18.96 ± 16.24	11.48	8.68 ± 7.27	5.14	6.12 ± 6.81	4.81	12.25	0.002**
	90	21.64 ± 16.06	11.36	10.17 ± 6.13	4.34	8.51 ± 5.94	4.20	26.80^a^	<0.001**
	85	24.75 ± 15.98	11.30	11.26 ± 6.05	4.28	9.23 ± 5.77	4.08	38.82^a^	<0.001**
	80	26.09 ± 17.91	12.66	13.14 ± 6.30	4.45	10.30 ± 6.32	4.47	30.55^a^	<0.001**
	75	29.77 ± 19.90	14.07	14.25 ± 7.44	5.26	11.17 ± 6.62	4.68	28.09^a^	<0.001**
	70	33.68 ± 21.00	14.85	16.84 ± 9.94	7.03	12.28 ± 5.50	3.89	29.55^a^	<0.001**
	65	36.67 ± 20.29	14.35	19.60 ± 10.11	7.15	13.61 ± 5.59	3.96	35.18^a^	<0.001**
	60	40.16 ± 20.71	14.64	22.62 ± 10.42	7.37	15.31 ± 6.07	4.29	34.64^a^	<0.001**
	55	44.16 ± 21.84	15.44	27.07 ± 12.16	8.60	17.01 ± 6.95	4.91	34.64^a^	<0.001**
	50	48.21 ± 21.45	15.17	30.25 ± 12.81	9.06	19.01 ± 8.31	5.87	36.36^a^	<0.001**
	45	49.96 ± 21.48	15.19	33.67 ± 14.46	10.22	20.59 ± 9.68	6.84	34.64^a^	<0.001**
	40	51.78 ± 20.71	14.64	36.25 ± 15.74	11.13	22.97 ± 10.76	7.61	34.64^a^	<0.001**
	35	54.30 ± 20.99	14.84	38.67 ± 16.25	11.49	25.25 ± 11.92	8.43	49.19^b^	<0.001**
	30	57.58 ± 20.93	14.80	42.01 ± 17.35	12.27	28.07 ± 12.62	8.93	59.11^b^	<0.001**
	25	57.81 ± 19.09	13.50	44.90 ± 17.79	12.58	29.73 ± 13.26	9.38	34.38^a^	<0.001**
	20	61.63 ± 18.95	13.40	45.68 ± 16.11	11.39	30.94 ± 11.18	7.91	36.29^a^	<0.001**
	15	63.37 ± 18.48	13.06	50.24 ± 18.56	13.12	33.98 ± 14.22	10.05	34.38^a^	<0.001**
	10	58.79 ± 20.20	14.29	51.20 ± 16.76	11.85	36.65 ± 14.41	10.19	18.11^a^	<0.001**
	5	53.85 ± 10.83	7.66	51.39 ± 17.18	12.14	40.23 ± 16.14	11.41	10.36^a^	0.006**
	0	45.05 ± 10.66	7.54	48.37 ± 21.81	15.42	42.24 ± 15.01	10.61	0.01^a^	1.00
DS	100	15.20 ± 11.60	8.20	6.34 ± 5.55	3.93	0.33 ± 0.47	0.33	4.00^a^	0.14
	95	17.54 ± 12.84	9.08	12.26 ± 12.94	9.15	6.51 ± 4.56	3.23	10.36^a^	0.006**
	90	18.04 ± 12.84	9.08	11.39 ± 8.08	5.72	6.60 ± 4.12	2.91	38.10^a^	<0.001**
	85	19.84 ± 11.06	7.82	12.17 ± 8.19	5.79	7.27 ± 4.12	2.91	38.27^a^	<0.001**
	80	22.19 ± 12.64	8.94	13.38 ± 9.11	6.44	8.00 ± 4.83	3.41	35.89^a^	<0.001**
	75	26.01 ± 15.64	11.06	15.1 ± 10.44	7.38	8.59 ± 5.15	3.64	36.64^a^	<0.001**
	70	30.61 ± 17.31	12.24	18.10 ± 12.41	8.77	9.89 ± 6.68	4.73	36.64^a^	<0.001**
	65	35.09 ± 17.13	12.12	21.50 ± 14.09	9.96	11.26 ± 7.04	4.98	36.64^a^	<0.001**
	60	38.10 ± 16.42	11.61	24.23 ± 14.54	10.28	13.02 ± 7.61	5.38	38.27^a^	<0.001**
	55	41.05 ± 16.87	11.93	26.98 ± 13.48	9.53	14.96 ± 8.12	5.74	71.75^b^	<0.001**
	50	44.93 ± 18.26	12.91	29.66 ± 14.26	10.08	17.17 ± 8.14	5.76	36.64^a^	<0.001**
	45	47.72 ± 16.92	11.96	32.48 ± 15.09	10.67	18.57 ± 8.82	6.23	89.95^b^	<0.001**
	40	48.52 ± 15.43	10.91	35.45 ± 16.10	11.38	20.73 ± 9.29	6.57	82.08^b^	<0.001**
	35	49.57 ± 15.26	10.79	37.50 ± 15.69	11.09	22.58 ± 9.34	6.60	67.53^b^	<0.001**
	30	50.52 ± 14.68	10.38	40.14 ± 14.14	10.00	23.82 ± 8.28	5.86	62.27^b^	<0.001**
	25	52.05 ± 13.44	9.50	40.22 ± 12.93	9.14	25.52 ± 8.10	5.73	54.47^b^	<0.001**
	20	52.39 ± 11.65	8.24	41.24 ± 13.15	9.30	26.92 ± 7.38	5.22	34.30^a^	<0.001**
	15	57.55 ± 13.03	9.22	42.24 ± 13.37	9.45	28.71 ± 8.10	5.73	32.40^a^	<0.001**
	10	53.90 ± 9.73	6.88	43.44 ± 13.35	9.44	33.69 ± 10.23	7.23	20.57^a^	<0.001**
	5	58.82 ± 12.43	8.79	46.88 ± 17.28	12.22	36.67 ± 12.69	8.97	9.49^b^	<0.001**
	0	56.39 ± 19.85	14.03	42.92 ± 20.15	14.25	39.79 ± 13.45	9.51	1.83^b^	0.25

NDS, non-dominant side; DS, dominant side; SD, standard deviation; CV, coefficient of variation; MVC, maximum voluntary contraction.

a Friedman’s test.

b Repeated-measures ANOVA.

** *p* ≤ 0.05 after Holm–Bonferroni correction.

Muscle activity across all conditions (60, 90, and 180°/s) on the isokinetic dynamometer on both the NDS and DS is presented in Table 6. The isokinetic dynamometer's muscle activation values showed no statistically significant differences (*p* > 0.05) between conditions on the NDS and across the majority of the ROM on the DS, indicating that the muscle activation generated by this system is not velocity dependent. The variability analysis revealed higher CV values compared with the VMAC®. These were always outside the acceptable range (>15%), with slightly greater values during full extension (0°).

**Table 6:** Descriptive statistics, variability, and between-conditions comparison (60, 90, and 180°/s) of muscle activity values across knee joint angles on both sides on the isokinetic dynamometer.

Side	Joint angle (°)	60°/s		90°/s		180°/s		Relationship between conditions	
		Mean ± SD	CV	Mean ± SD	CV	Mean ± SD	CV	Test	*p-*Value
		(% MVC)	(%)	(% MVC)	(%)	(% MVC)	(%)		
NDS	100	49.49 ± 22.75	45.96	45.86 ± 21.10	46.01	53.03 ± 17.08	32.21	0.51^a^	0.61
	95	50.53 ± 22.32	44.17	51.04 ± 23.76	46.56	52.93 ± 17.40	32.88	0.26^a^	0.77
	90	51.34 ± 21.53	41.94	54.59 ± 23.65	43.33	55.58 ± 18.63	33.51	0.74^a^	0.48
	85	52.05 ± 21.36	41.05	55.61 ± 22.15	39.83	56.87 ± 18.61	32.72	0.99^a^	0.38
	80	53.64 ± 20.73	38.65	56.70 ± 22.04	38.87	57.58 ± 18.77	32.61	0.60^a^	0.55
	75	55.20 ± 20.05	36.31	58.18 ± 21.74	37.37	59.05 ± 19.40	32.85	0.54^a^	0.59
	70	54.76 ± 19.63	35.85	59.97 ± 21.26	35.46	59.70 ± 19.48	32.64	0.36^b^	0.83
	65	55.15 ± 19.78	35.88	61.00 ± 20.83	34.15	61.64 ± 20.01	32.47	0.27^b^	0.87
	60	57.00 ± 20.37	35.74	62.38 ± 21.27	34.09	62.14 ± 20.65	33.23	0.09^b^	0.96
	55	58.52 ± 20.43	34.92	63.33 ± 21.06	33.25	62.41 ± 20.81	33.35	1.18^b^	0.55
	50	59.71 ± 20.12	33.70	64.35 ± 19.79	30.76	62.57 ± 20.69	33.07	0.36^b^	0.83
	45	61.36 ± 19.80	32.27	64.78 ± 18.91	29.19	62.34 ± 20.02	32.11	1.91^b^	0.38
	40	62.98 ± 21.59	34.29	63.98 ± 17.82	27.85	61.78 ± 19.71	31.91	0.64^b^	0.73
	35	63.57 ± 23.36	36.75	62.16 ± 16.94	27.25	60.41 ± 18.82	31.16	0.32^a^	0.65
	30	62.18 ± 23.48	37.76	59.77 ± 16.21	27.12	59.16 ± 18.76	31.71	0.30^a^	0.74
	25	60.47 ± 22.83	37.76	59.00 ± 18.05	30.59	57.35 ± 18.72	32.64	0.29^a^	0.75
	20	58.12 ± 21.69	37.31	57.77 ± 18.96	32.81	56.40 ± 18.65	33.06	0.11^a^	0.90
	15	54.12 ± 21.55	39.82	55.84 ± 18.62	33.34	53.82 ± 21.29	39.56	0.82^b^	0.66
	10	50.47 ± 18.52	36.69	53.89 ± 16.98	31.52	52.58 ± 20.75	39.47	0.69^a^	0.51
	5	47.89 ± 16.69	34.86	51.28 ± 14.90	29.07	51.77 ± 18.62	35.96	0.38^a^	0.69
	0	46.48 ± 16.98	36.54	54.38 ± 20.37	37.46	51.17 ± 19.42	37.96	0.22^a^	0.80
DS	100	43.89 ± 23.14	52.74	44.21 ± 21.91	49.56	51.79 ± 23.52	45.41	2.68^a^	0.08
	95	47.31 ± 23.60	49.89	46.97 ± 21.01	44.74	52.15 ± 22.09	42.36	1.53^a^	0.23
	90	49.08 ± 22.77	46.38	49.44 ± 19.98	40.42	53.48 ± 20.36	38.06	1.30^a^	0.28
	85	50.14 ± 20.75	41.39	51.23 ± 20.25	39.52	54.74 ± 19.27	35.20	1.23^a^	0.30
	80	51.13 ± 18.12	35.45	51.91 ± 20.49	39.48	55.32 ± 18.44	33.33	1.12^a^	0.34
	75	52.00 ± 16.98	32.66	52.58 ± 20.78	39.51	56.09 ± 17.65	31.47	1.11^a^	0.34
	70	52.81 ± 16.47	31.19	53.05 ± 20.47	38.58	55.91 ± 17.24	30.84	0.66^a^	0.52
	65	53.20 ± 16.45	30.93	53.96 ± 19.70	36.51	55.79 ± 17.12	30.69	3.27^b^	0.20
	60	54.03 ± 16.92	31.32	55.61 ± 19.55	35.15	54.73 ± 16.62	30.37	1.18^b^	0.55
	55	55.32 ± 17.72	32.04	57.34 ± 18.12	31.61	53.65 ± 17.21	32.07	0.64^b^	0.73
	50	57.63 ± 18.14	31.48	57.95 ± 16.08	27.76	52.83 ± 17.72	33.55	2.17^a^	0.13
	45	59.49 ± 16.87	28.35	58.38 ± 15.50	26.56	51.75 ± 17.95	34.68	5.02^a^	0.01*
	40	59.86 ± 16.33	27.29	57.97 ± 16.32	28.15	51.33 ± 18.58	36.20	6.10^a^	0.01*
	35	59.80 ± 16.67	27.88	57.42 ± 18.34	31.94	50.90 ± 19.37	38.05	5.93^a^	0.01*
	30	60.13 ± 18.40	30.59	56.95 ± 19.94	35.02	50.60 ± 19.68	38.90	6.46^a^	0.01*
	25	59.65 ± 21.14	35.44	54.83 ± 21.40	39.04	50.48 ± 19.80	39.22	11.27^b^	0.01*
	20	58.20 ± 23.66	40.66	51.26 ± 21.30	41.54	51.18 ± 20.08	39.24	3.63^a^	0.04*
	15	55.99 ± 23.68	42.30	47.84 ± 20.31	42.45	51.49 ± 20.57	39.94	4.01^a^	0.03*
	10	53.28 ± 21.68	40.69	46.17 ± 19.78	42.84	55.83 ± 17.98	32.20	1.13^b^	0.57
	5	51.29 ± 20.11	39.21	44.56 ± 19.37	43.47	55.17 ± 17.48	31.69	3.57^b^	0.17
	0	47.82 ± 20.43	42.72	56.85 ± 18.10	31.83	54.52 ± 17.75	32.56	5.09^b^	0.08

NDS, non-dominant side; DS, dominant side; SD, standard deviation; CV, coefficient of variation; MVC, maximum voluntary contraction.

a Repeated-measures ANOVA.

b Friedman’s test.

* *p* ≤ 0.05 (uncorrected).

On the NDS, across all conditions, the VMAC® system's muscle activation values showed a progressive increase from the initial-ROM joint angles (100°) to the mid-ROM joint angles (approximately 50°), followed by a less pronounced increase toward the final-ROM joint angles (0°). In contrast, the isokinetic dynamometer's muscle activation values remained almost unaltered across the entire ROM.

The DS muscle activation curves from the VMAC® system were initially lower, pronouncedly increasing toward the mid-ROM joint angles (45°) and then less pronounced from the middle to final ROM joint angles (45°–0°). Across all conditions, the isokinetic dynamometer's muscle activation curves showed a relatively stable curve across the entire ROM.

## 4 Discussion

The present study aimed to analyze the performance of the VMAC®, examining torque production and muscle activation across the full ROM (100°–0°) at 5° intervals. The main findings of this study were as follows: (i) the VMAC®'s torque values closely aligned with the torque values obtained on the isokinetic dynamometer across the initial and mid-ROM joint angles; (ii) the torque values obtained with the VMAC® replicated the inverted U-shape strength curves observed on the isokinetic dynamometer across the ROM, with a misalignment at the final ROM joint angles; (iii) the torque measurements obtained with the VMAC® demonstrated minimal between-session variability, reinforcing the device's reliability; (iv) the muscle activation patterns assessed on the VMAC® differed from those observed on the isokinetic dynamometer due to differences in loading intensity, as the repetitions on the VMAC® were performed at a controlled angular velocity and the repetitions on the isokinetic dynamometer were performed at maximum effort. The defined hypotheses were partially attained.

The torque data revealed that the isokinetic dynamometer and the VMAC® produced similar values and the expected inverted U-shaped strength curve, with peak values occurring at around 65° of knee flexion, similar to other studies that also identified this pattern (7, 8, 11, 12, 18). Nevertheless, the VMAC®’s observed values only partially matched the strength curves obtained with the isokinetic dynamometer, specifically, at the initial and middle angles of the ROM (100°–35°), despite the efforts to generate a variable cam system that matches the HTC across the entire ROM. This has also been noted in the literature, as several authors have referenced the inability of variable cam systems to match the HTC at the initial and final joint angles of the ROM and highlighted the need to better align the MRT with the HTC (7, 11, 12). However, the VMAC® attained more matched strength curves when compared to those in the literature, achieving similar values to the HTC at the final joint angles of the ROM and matching the HTC at the initial joint angles of the ROM.

In a previous study by da Costa-Machado et al. (18), the VMAC®'s torque production was tested isometrically at 100°, 80°, 60°, 40°, 20°, and 0° and compared with an isokinetic dynamometer's torque values. The results suggested a match across the entire ROM. However, these tests were performed in isometry and at maximum capacity, which differs from our study, in which concentric values from the VMAC® were generated at a controlled angular velocity and not at maximum effort.

In our study, the CV values obtained using the VMAC® were mainly below the 5% mark and always within the acceptable range of 15%, which demonstrates excellent reproducibility and the internal consistency of the equipment. This does not follow the literature, where the initial and final joint angles of the ROM are expected to demonstrate a higher variability associated with unfavorable muscle length–tension relationships, reduced effective moment arms, increased passive tissue contributions, and altered neural drive, all of which may reduce force stability. In contrast, the CV values obtained on the isokinetic dynamometer were considerably higher. This difference was expected, as the VMAC® measurements were collected under submaximal loading, whereas the dynamometer values arose from maximal voluntary efforts, conditions that naturally influence variability and amplify fluctuations in torque output.

The observed muscle activity curves differ from those observed by Häkkinen et al. (1), who reported a steady initial to mid-ROM muscle activity curve and a descending pattern toward the full extension. However, they are similar to the muscle activation patterns observed by Aboodarda et al. (19, 20), who reported progressively increasing muscle activity through the ROM. In our study, the 20 and 30 kg conditions on the VMAC® achieved a similar progressively increasing muscle activation pattern despite the magnitude differences. The 40 kg condition achieved a slightly inverted U-shape muscle activation pattern, possibly due to the higher load that further enhanced the difficulty in the final ROM angles. Häkkinen et al. (1) performed the test using a David F 200 leg extension machine (David F 200, David International Ltd., Helsinki, Finland), which provides a different angle–torque relationship from that observed on the VMAC®; thus, the discrepancy in the muscle activity curves may be due to this. Aboodarda et al. (19) performed their assessments on a Nautilus leg extension machine, which produces a strength curve that differs completely from the VMAC®, presenting with an almost constant value across the entire ROM and a slight decrease at the mid-ROM (20). This is different from the inverted U-shape curve observed on the VMAC®.

Moreover, the muscle activity values obtained on the VMAC® differed from those obtained on the isokinetic dynamometer. However, the assessment on the VMAC® was performed at a controlled velocity with a non-maximal load, in contrast to the maximum effort on the isokinetic dynamometer. This explains the similarity in the torque values and the discrepancy in the muscle activity values.

These results further underscore that torque and muscle activity do not represent the same construct. Torque reflects the combined effects of force, moment arm geometry, and joint mechanics, whereas muscle activity captures neural drive and recruitment behavior, which does not necessarily rise in parallel with mechanical output.

## 5 Limitations

This study has several limitations that should be acknowledged, namely, (i) torque and muscle activity responses on the VMAC® were not examined under high load conditions; (ii) muscle activation was recorded from only one quadriceps muscle, which restricts the ability to generalize activation patterns across the entire muscle group; (iii) the relatively large variability in the muscle activity signals reflects the well-known challenges associated with surface muscle activity measurements; (iv) the reduced sample size, participant characteristics, and lack of prospective sample size calculation or agreement-based sample size estimation may limit broader applicability, as trained athletes or clinical populations could display different torque–activation relationships.

Future studies analyzing this equipment should assess more muscle groups (agonists, antagonists, and synergists). Female participants and subjects with distinct levels of physical activity and from different sports should also be included. Finally, considering the final-ROM joint angle misalignment, further improvements to the VMAC® should be encouraged so that the MRT matches the HTC across the entire ROM.

## 6 Conclusions

The VMAC® system produced strength curves that were generally consistent with those of an isokinetic dynamometer, with the strongest agreement at mid-ROM joint angles and higher divergence at the final-ROM joint angles. Muscle activity patterns were similar across conditions, though the variability was higher than that for torque. These findings appear to support the VMAC® as a viable method for replicating the HTC strength curve and highlight opportunities to refine cam geometry for improved angle-specific resistance.

## 7 Patents

Patent pending under the number 121049 at https://inpi.justica.gov.pt/.

## Data Availability

The raw data supporting the conclusions of this article will be made available by the authors, without undue reservation.
